# A Media-Based School Intervention to Reduce Sexual Orientation Prejudice and Its Relationship to Discrimination, Bullying, and the Mental Health of Lesbian, Gay, and Bisexual Adolescents in Western Canada: A Population-Based Evaluation

**DOI:** 10.3390/ijerph15112447

**Published:** 2018-11-02

**Authors:** Jillian Burk, Minjeong Park, Elizabeth M. Saewyc

**Affiliations:** Stigma and Resilience Among Vulnerable Youth Centre, School of Nursing, University of British Columbia, Vancouver, BC V6T 2B5, Canada; jillianburk@gmail.com (J.B.); minjeong.park@alumni.ubc.ca (M.P.)

**Keywords:** adolescent, LGBT youth, school intervention, bullying, mental health

## Abstract

School interventions to address sexual orientation discrimination can be important tools for fostering inclusive school climate, and improving student wellbeing. In this study, we empirically evaluated a film-based intervention, Out in Schools, designed to reduce sexual orientation prejudice and foster inclusive school attitudes. Our evaluation mapped data about Out in Schools presentations onto student data from the random cluster-stratified, province-wide 2013 British Columbia Adolescent Health Survey (BCAHS) as well as potential confounding variables of Gay-Straight Alliance clubs (GSAs) and inclusive school policies. Outcome measures included past year sexual orientation discrimination, bullying, suicidal ideation, and school connectedness among lesbian, gay, and bisexual (LGB) and heterosexual (HET) students in grades 8 through 12 (ages 13 to 18; unweighted *N* = 21,075, weighted/scaled *N* = 184,821). Analyses used complex samples logistic regression, adjusted for sample design, conducted separately by gender and orientation. We found Out in Schools presentations were associated with reduced odds of LGB students experiencing discrimination, and both LGB and HET girl students being bullied or considering suicide, and increased levels of school connectedness, even after controlling for GSAs and policies. Out in Schools appears to have an additive contribution to reducing orientation prejudice and improving LGB and heterosexual student wellbeing within schools.

## 1. Introduction

There are many well-documented health disparities between lesbian, gay, and bisexual (LGB) adolescents and their heterosexual peers, especially in regards to mental health. Systematic reviews and meta-analyses have consistently found that LGB youth have a higher prevalence of depression, self-harm, suicidal ideation and attempts, and problematic substance use, compared to heterosexual youth [1,2,3,4]. In Canada-specific reviews and population-based studies, similar results have been found: Canadian LGB youth are significantly more likely to report substance use, psychological distress, and suicidal behaviours [5,6,7] with persistent trends in these disparities [8,9].

Furthermore, LGB students experience differential social outcomes, particularly within the school environment. These students report more hostile school climates and decreased feelings of safety at school, and are more likely to skip school and have poorer academic achievement as a result [10,11,12]. Research in the United States and Canada has demonstrated that sexual minority students are significantly more likely to experience bullying, victimization, and violence from their peers, and homophobic discrimination from both peers and teachers [10,13,14]. This increased bullying and victimization has been directly linked to the observed disparities in mental health outcomes. Numerous studies have shown that homophobic bullying in schools is a mechanism for adverse health among LGB youth, and a mediating factor for experiencing increased depression, suicidality, and substance use [12,15,16,17]. The effects of school-based homophobic bullying can have a lasting influence, and negatively impact psychosocial adjustment and risk-taking behaviours into young adulthood [18].

While bullying and victimization are negative factors associated with health disparities, research has shown that supportive school climates and school-based initiatives to reduce bullying serve as protective factors against these inequities [19,20,21]. In order to improve school climate and mitigate negative outcomes for sexual minority adolescents, several school-based interventions have been developed and implemented. These include supportive clubs for lesbian, gay, bisexual, transgender, queer and questioning (LGBTQ) students, like Gay-Straight Alliances or Gender-Sexuality Alliances (GSAs), inclusive anti-bullying policies that specifically reference sexual orientation and gender identity, and inclusive school curricula that address sexual orientation and gender identity (SOGI). Population-based research and systematic reviews have demonstrated that GSAs are associated with positive school environments and better health. These clubs have been found to reduce the likelihood of bullying, substance use, and suicidality for all students, regardless of sexual orientation or whether students were actual club members [6,7,22,23]. Additionally, evidence-based anti-bullying policies and LGB inclusive curriculum materials have similar effects in improving student mental health and academic achievement, and fostering a better school climate, while reducing bullying [10,23,24,25]. However, such policies must be fully implemented and enforced by staff members in order to maintain effectiveness, an aspect rarely included in their evaluation [26].

As the beneficial effects of GSAs and LGBTQ-inclusive policies have become better documented, several Canadian provinces have introduced legislation or regulations to incorporate this knowledge and reduce harmful bullying. This includes legislation in the last few years to protect students’ rights to create GSA clubs, as well as developing SOGI curricula, policies, and resources for educators [27,28,29]. These evidence-based effective approaches are becoming more prevalent in Canada, yet analyses of trends in LGB health suggest the gaps in health still remain, or are only slowly improving [8,9].

It is important, therefore, to find other strategies that can help to further reduce health disparities among students. Other school-based strategies could include media-based interventions to reduce discrimination, as research has shown that favorable, multi-dimensional media and cultural representations of LGB individuals positively influence resiliency, self-perception, and social support among LGB youth [30]. A number of studies over the past three decades have examined intervention strategies such as panel discussions, theatre performances, or films about LGBTQ people, combined with discussions guided by LGBTQ facilitators, to reduce homophobic attitudes and prejudice, foster empathy and increase supportive attitudes, and potentially to change stigma behaviours. Although panel discussions and informational presentations have shown mixed results in reducing homophobic attitudes [31,32] interventions that involve theatre performances have shown significant, and in some cases, sustained reduction in homophobic attitudes [33,34]. Interventions using films have also shown positive effects [31,32,35].

These performance-based or media-based approaches have been based in part on Intergroup Contact Theory [36], which postulates that structured exposures to people from a stigmatized or marginalized group can reduce negative stereotypes and prejudice; and in part on the use of Theatre for Social Justice [33], a form of educational performance designed to teach about difference and elicit positive discussions about social justice. In Theatre for Social Justice, the primary theorized mechanisms of effect for are not informational or cognitive, but emotional. Whether in the storytelling of panels (which often include the coming out stories of the panelists), in theatre performances, or in films about LGBTQ people, drama “evokes emotional responses and holds the potential to leave powerful impressions with an audience” (p. 44). With the exception of one study of a theatre performance combined with curriculum integrated across a variety of high school courses [34], most of these interventions have been tested only with college students, not younger adolescents. As well, many of the interventions focused on short-term attitude changes within individuals, rather than the potential for there to be broader effects of a shift in attitudes and behaviours within a school climate.

### The Out in Schools Film-Based Intervention

One example of a film-based program to reduce homophobia, biphobia and transphobia is Out in Schools, a school-based initiative, operated in the westernmost Canadian province of British Columbia (BC), by the non-profit organization, Out on Screen. Founded in 2004, Out in Schools presents LGBTQ film screenings and hosts facilitated group dialogues about the films afterwards, discussing themes of gender, sexuality, and LGBTQ lived experiences. Their goal is to engage students and teachers on issues of homophobia, biphobia, transphobia, and bullying, to promote safer and more inclusive learning environments [37]. A typical Out in Schools presentation involves a series of short films, introduced and punctuated by guided discussion about key concepts related to the diversity of genders and sexual orientation, issues of discrimination and bullying, and ways to be inclusive. The films tend to be teen-oriented, tell engaging stories about young people, often with humorous or emotionally touching content, and feature ethnically and culturally diverse protagonists to fit the diversity among students in BC schools. An event is facilitated by 1 to 3 adults who identify as LGBTQ+ and are trained in dynamic speaking and developmentally appropriate content for children as young as grades 5 and 6 (ages 10 to 11) up to grades 12 (ages 17 to 18). Each event will last from 1 to 2 h, and will feature a combination of films ranging from 1 to 10 min, from 11 to 20 min, and sometimes a film longer than 20 min. Table 1 provides a list of example titles of films shown by Out in Schools during 2007 to 2013.

Out in Schools events are booked by schools for small showings, for example, to a GSA group, or to a class of approximately 30 students during class time. They can also be booked for a larger audience of students, in auditorium showings for between 100 and 250 students. They also offer larger schools the option of 2 to 5 showings on the same day to different classrooms or groups of students. Several schools have had showings each year, or events more than once a year. During 2009 and 2010, Out in Schools had a separately funded project in which filmmakers visited schools to help classes create brief public service announcements (PSAs) against homophobia and bullying, or create positive messages supporting LGBTQ+ students. The PSAs scripted by, acted in, and filmed by the students were then entered into an annual contest. In subsequent years, several of the winning PSAs were included in the Out in Schools film events, based on the theory that students would be even more engaged with content that they knew was created by fellow students.

Although Out in Schools has regularly distributed brief feedback forms at the end of most showings and discussion sessions, there has not been a more rigorous, independent evaluation of the potential contributions of Out in Schools to improving school climate and fostering school connectedness to support positive mental health for LGBTQ and heterosexual youth. Out in Schools staff approached the Stigma and Resilience Among Vulnerable Youth Centre, a multidisciplinary research centre at the University of British Columbia, to request an evaluation.

Thus, the purpose of this study was to evaluate the Out in Schools film-based intervention and its association with mental health outcomes and bullying experienced by sexual minority adolescents. We assessed the relationship between LGB and heterosexual students’ experiences of suicidal ideation, bullying and discrimination, and school connectedness in schools hosting the Out in Schools presentations, compared to LGB and heterosexual students in schools that did not host the events. We hypothesized that where schools had hosted Out in Schools presentations, students would report better mental health outcomes, lower levels of bullying, and higher school connectedness, after controlling for other potential supports, such as the presence of GSAs or LGBTQ-inclusive school policies.

## 2. Materials and Methods

This study was a retrospective evaluation of the Out in Schools program through the first half of 2013, drawing on secondary analysis of the 2013 British Columbia Adolescent Health Survey (BCAHS) as a population-level source of evidence. The BCAHS was a voluntary, anonymous youth health survey, administered to students in grades 7–12, in 1643 classrooms across 56 of the 59 school districts in British Columbia, (*N* = 29,832). BCAHS data were weighted to adjust for differential probability of selection, response rates, and proportion of enrollment, and represents 98.5% of students enrolled in public schools in the province. Survey procedures were approved by the Behavioural Research Ethics Board of the University of British Columbia (#H12-02630).

The analytic sample for the current study was limited to those students who had identified as lesbian, gay, or bisexual (LGB) or completely heterosexual (HET) on a sexual orientation measure that combined both attraction and labels. The response options for this measure included “completely heterosexual (attracted to the opposite sex)”, “mostly heterosexual”, “bisexual (attracted to both men and women)”, “mostly homosexual”, “completely homosexual (gay or lesbian; attracted to my same sex)”, “questioning”, and “I don’t have attractions”. For this study, we selected bisexual, mostly homosexual, and completely homosexual participants, who were aggregated to form the LGB group, and those who identified as completely heterosexual for the HET group, as previous research has shown some school-based interventions to support LGB youth appear to have benefits for heterosexual youth as well [6,7]. We further limited the sample to students who had provided a valid answer to a question about their gender, as analyses were conducted separately by gender, given gender differences in bullying and in mental health outcomes. Finally, the sample was limited to students in grades 8 through 12, because in 2013, elementary schools in BC, including grade 7, did not have GSAs [7]. The final sample contained 998 LGB students and 20077 HET students, which were weighted to adjust for the differential probability of selection in different regions, and scaled to represent the enrolled population in the participating schools, *N* = 184,821; see Table 2 for demographic characteristics of the sample.

Out in Schools provided a list of their presentations from their inception in 2004 through to 2014. Information included host school, school location (city and school district), and date of the presentation. From its inception in 2004, through to 2014, Out in Schools hosted 275 school-based events (415 screenings, as some days they held four screenings in a single school for different grades). These presentations were hosted in 28 of the 59 school districts, in 113 different schools or school programs, and 49.6% (*n* = 56) of those schools held at least two events (range 1 to 14 events per school, with between 1 and 5 screenings at each event). Out in School presented increasing numbers of events over the years, beginning with only three events (three screenings) in 2004 increased to 18 events (36 screenings) in 2009, to 57 events (92 screenings) in 2013. Some of the schools that hosted Out in Schools events were not among the randomly selected schools in the 2013 BCAHS; thus, in the final analytic sample for this study, there were a total of 240 sampled schools, and Out in Schools had presented events in 61 of those schools (25.4%).

### 2.1. Intervention Measures

Three separate measures were developed from the Out in Schools presentations data to test as explanatory variables. The first variable was a dichotomous indicator of whether or not a student attended a school that had ever hosted an Out in Schools presentation. The second measure was the total number of Out in Schools presentations that a particular school had hosted since the program began in 2004, to measure cumulative exposure on school climate over time. The final measure was another cumulative exposure variable, except only the Out in Schools presentations that occurred from 2009 to 2013 were included. This time period corresponds to the same years in which students taking the 2013 BCAHS survey would have been in high school, thus measuring potential direct exposure during the survey cycle, and more proximal climate effects.

### 2.2. Outcome Variables

Four primary categories of potential outcomes were evaluated in the models for their hypothesized relationship to Out in Schools events:

*Homophobic Discrimination:* Student experiences with discrimination based on perceived sexual orientation were measured with a single survey question: “In the past 12 months, have you been discriminated against or treated unfairly because of your sexual orientation (being or thought to be gay or lesbian)?” Responses were “yes” or “no”.

*Bullying:* Bullying amongst peers was measured with two items, one addressing verbal bullying and one for relational bullying. Teasing or verbal harassment is a method of direct verbal bullying, whereas social exclusion is a method of indirect relational bullying [38]. Teasing was measured with the item, “During the past 12 months, while at school or on the way to and from school, how many times did another youth tease you or say something personal about you that made you feel bad or extremely uncomfortable?” Exclusion was measured with the item, “During the past 12 months, while at school or on the way to and from school, how many times did another youth keep you out of things on purpose, exclude you from their group of friends, or completely ignore you?” Both questions had the potential responses, “Never”, “Once”, “Twice”, or “3 or more times”, but for analytic purposes in this study, the responses were aggregated to either “Never” or “One or more times”.

*Suicidality:* Suicidal ideation was measured with one binary item: “During the past 12 months, did you ever seriously consider killing yourself (attempting suicide)?” Response options were “yes or “no”.

*School Connectedness:* School connectedness was assessed with two correlated sub-scales relating to school belonging and teacher connectedness, each with three statements. For school belonging, students were asked whether they felt like they were a part of school, whether they were happy to be at their school, and whether they felt safe at school. For teacher connectedness, students were asked whether they felt school staff treated them fairly, whether their teachers cared about them, and whether other school staff cared about them. Responses were on a scale of 1 to 5, from strongly disagree, to strongly agree. To determine the score for each sub-scale, responses were averaged across the three corresponding questions.

### 2.3. Confounding Variables

Additional variables were included in the analyses to improve the effect measure estimates. Student grade level, urban/rural school location, and presence of a GSA or anti-homophobia school policy were also included as confounding variables in analytic models. All schools that participated in the 2013 BCAHS were telephoned in 2013 and 2014 to identify whether their school had a GSA and/or school policy that specifically addressed bullying toward LGBTQ students, and what year these had been implemented [7]. The majority of students lived in urban areas (90% vs. 10% rural areas).

### 2.4. Data Analyses

Out in Schools presentation data from April 2004 through June 2013 were mapped onto the students in the relevant school in the BCAHS dataset, and analyses were conducted using SPSS version 24 Complex Samples (IBM, Armonk, NY, USA). Logistic regressions were performed for dichotomous outcome variables (suicidality, discrimination, teasing, and exclusion), and linear regressions were conducted for the continuous variable, school connectedness, to assess effects. We used an alpha value of 0.05 to determine statistical significance. Analyses were conducted separately for boys and girls, as previous research has shown differential health outcomes across genders, within populations of bisexual and homosexual adolescents [39]. As mentioned above, analyses were also adjusted for the presence of a GSA and/or anti-homophobia policy in school, grade level, and urban or rural school location.

## 3. Results

Table 3 presents the results for boys and girls for any exposure to Out in Schools events. In schools where Out in Schools had hosted at least one presentation, LGB and heterosexual girls reported significantly better outcomes than in schools that had never hosted an Out in Schools event. Where Out in Schools had ever been present in their school, lesbian and bisexual girls had significantly lower odds of reporting homophobic discrimination, considering suicide, or being verbally bullied, compared to their peers who attended schools that did not host Out in Schools events. Similarly, heterosexual girls in schools with any Out in Schools events had significantly lower odds verbal harassment and social exclusion, as well as suicidal ideation. Gay and bisexual boys were significantly less likely to experience verbal bullying in schools that had hosted Out in Schools events, with nearly half the odds of being teased; however, for heterosexual boys, there was no relationship between Out in Schools events and discrimination, bullying, or suicidal thoughts.

School connectedness measures showed varying effects by orientation group: gay and bisexual boys had no significant difference in either school belonging or teacher connectedness in schools with Out in Schools presentations compared to those with no events. Lesbian and bisexual girls reported lower school belonging scores, on average, in Out in Schools host schools; the presence of one or more Out in Schools events were associated with a 0.15 decrease in the average school belonging score. In contrast, both heterosexual boys and girls reported significantly higher teacher connectedness in schools that had one or more Out in Schools events, although there were no significant effects for school belonging.

When adjusting for the potentially confounding presence of a GSA or anti-homophobic bullying policy within schools, many of these results persisted for lesbian, bisexual, and heterosexual girls, but not often for boys (also Table 3). In regression models controlling for confounding variables, lesbian and bisexual girls had one-third the odds of seriously considering suicide or experiencing homophobic discrimination, and gay and bisexual boys had half the odds of being verbally bullied in schools that had hosted Out in Schools, compared to schools that had not. After adjusting for confounding variables, there was no longer a significant decrease in school belonging associated with Out in Schools presentations for lesbian and bisexual girls. Among heterosexual students, after controlling for the presence of GSAs and anti-homophobia policies, heterosexual girls still had 12% lower odds of social exclusion in the past year, and 17% lower odds of suicidal ideation, as well as a significant increase in teacher connectedness. Associations for heterosexual boys were no longer significantly different.

Table 4 shows the results of repeated exposures to Out in Schools events within schools. With each subsequent Out in Schools presentation that a school hosted, there were further effects on student outcomes. When considering the cumulative effect of Out in Schools since its inception, every additional presentation was associated with a decrease in lesbian and bisexual girls reporting homophobic discrimination, verbal bullying, and suicidal ideation, and there was a significant increase in school connectedness scores. These findings were similar for completely heterosexual girls, with the number of performances in schools linked to significantly lower odds of sexual orientation discrimination, bullying, and suicidal ideation. In contrast, though, heterosexual girls reported lower teacher connectedness with increased numbers of repeat events in their school, although it should be noted the regression coefficients and R^2^ values are quite small. Among gay and bisexual boys, there were significantly lower odds of experiencing discrimination and verbal or relational bullying, as well as an increase in average teacher connectedness scores linked to repeated Out in Schools events. For heterosexual boys, there were still no associations between Out in School events and discrimination, bullying, or suicidality, and as with heterosexual girls, significantly lower teacher connectedness, although similarly, small coefficients and R^2^ values.

In schools that already had GSAs or anti-homophobia policies, there were still additive improvements from each additional presentation event. After controlling for confounding variables, lesbian and bisexual girls had 0.86 the odds of considering suicide with each subsequent presentation, compared to students in schools that had no presentations.

Heterosexual girls had lower odds of discrimination based on perceived sexual orientation, social exclusion, and suicidal thoughts. Gay and bisexual boys also appeared to have additional benefits with repeated presentations: the odds of experiencing homophobic discrimination or either form of bullying were still lower, and school connectedness scores increased with each presentation by 0.05 on average. For heterosexual boys, teacher connectedness scores decreased significantly with repeated presentations, although by only −0.01 on average.

Table 5 presents the evaluation of the cumulative effects only from presentations that have occurred between 2009 and 2013. There were similar significant effects from each subsequent presentation. After each additional Out in Schools presentation, the odds of lesbian and bisexual girls considering suicide or experiencing verbal bullying were significantly reduced, and their school belonging scores were significantly higher; heterosexual girls’ odds of considering suicide, verbal harassment, or social exclusion were lower, and their teacher connectedness scores were also lower, although with very small effect sizes. Gay and bisexual boys had reduced odds of experiencing verbal bullying, and their teacher connectedness scores increased with each presentation, compared to students whose schools did not host any Out in Schools events, but there were no significant effects for heterosexual boys, except a small decrease in teacher connectedness. Similar results were observed after controlling for the presence of GSAs and anti-homophobia policies: lesbian and bisexual girls had 16% lower odds and heterosexual girls had 6% lower odds of considering suicide with each event, gay and bisexual boys’ teacher connectedness scores were 0.08 higher, and heterosexual girls and boys had lower teacher connectedness scores, but with small coefficients.

## 4. Discussion

This study sought to evaluate the relationship between a film-based anti-discrimination program, Out in Schools, and LGB and heterosexual students’ well-being within schools in western Canada. The ability to retrospectively map the events onto population-based adolescent health data created an opportunity to evaluate an uncontrolled intervention, as a form of natural experiment, a potentially effective strategy for evaluating population health interventions where randomized trials are not feasible. We found that where schools had hosted Out in Schools, LGB students were less likely to have experienced bullying, discrimination, or to have seriously considered suicide in the past year, in addition to reporting higher levels of school connectedness. There were also associations with lower odds of bullying and suicidality for heterosexual girls, but not for heterosexual boys. Even in schools that already had GSAs and/or LGBTQ-inclusive policies, Out in Schools presentations were significantly associated with lower odds of LGB students reporting they had considered suicide, been bullied or been discriminated against in the past year, as well as increased teacher connectedness, and similar results for heterosexual girls, except for teacher connectedness. Notably, in Out in Schools host schools, lesbian and bisexual girls were one-third less likely to have experienced anti-LGBT discrimination or to have considered suicide, and gay and bisexual boys were nearly half as likely to have experienced relational bullying. We also found an additive effect of multiple exposures: with each repeated visit by Out in Schools, the odds of bullying, discrimination, or suicidality was further significantly lowered; for gay and bisexual boys, each presentation brought a 16% reduction in the odds of being teased or discriminated against, and increased teacher connectedness scores, while for lesbian and bisexual girls, each presentation led to a 14% decrease in the odds of suicidality, and for heterosexual girls, each presentation was linked to 5% lower odds of suicidality; given the cumulative effect of repeated events, this translates to a 20% reduction in odds of suicidal ideation among heterosexual girls in schools that had Out in Schools events every year for four years, which, given the large number of heterosexual girls, is not a trivial effect.

Our results support our initial hypothesis that students in Out in Schools host schools would report improved outcomes, and after ruling out competing explanations from other interventions such as GSAs, our findings indicate that Out in Schools may still have an additive contribution to reducing homophobia and bullying, and improving mental health among LGB adolescents, and heterosexual girls. As the proposed mechanism for these observed effects, we suggest such a program may have contributed to altering the overall school climate, which in turn exerted a positive effect on students’ well-being.

Even after adjusting for confounding variables, the majority of odds ratios and beta coefficients remained significant, with non-trivial effect sizes. While it is possible we might be missing other key program influences, by controlling for the most likely confounding influences of LGBTQ-related interventions, with limited attenuation of the results, our findings suggest that most of these effects could be attributed, at least in part, to Out in Schools. Our results also suggest an added benefit in integrating supplemental programs like Out in Schools into overall LGBTQ-inclusive school strategies.

The findings from this study align with the results in the literature about other, more well-established interventions that have been introduced into schools, such as GSAs, inclusive policies, and inclusive curricula [7,10,22] as well as previous research on theatre-based or film-based one-time events among college students [31,32,33,35]. Our study’s findings of similar effects in reducing bullying and negative mental health outcomes, while increasing school connectedness, were linked to a novel intervention to support LGB students; very few studies have been published that assess this type of film-based supplementary program, and none we have found focus on this type of program for high school students, nor for heterosexual students. Our research appears to be among the first evaluations of an innovative film-based school program for LGBTQ (and heterosexual) students.

### Limitations and Strengths

All studies have limitations that should be considered in assessing their relevance to other locations, as well as potential strengths. The primary limitation to this study is that, due to the retrospective nature of the evaluation and the use of existing survey data, we could not identify whether the students who were surveyed by the BCAHS had actually attended the Out in Schools presentations and so were directly affected by the program, nor could we measure change over time for individual students. Instead, we were limited to the assumption that Out in Schools events would contribute to a school effect, influencing the overall school climate, which in turn should affect student responses. Another limitation is the data were cross-sectional in nature, which impedes the ability to infer causality in the relationship between Out in Schools events and student outcome measures. At the same time, it is difficult to randomly assign population health interventions across enough schools to adequately power such an intervention study, and the costs of such repeated measures data collection before and after exposure to an intervention in so many schools across the province also renders it financially infeasible. However, by controlling for the presence of GSAs or LGBTQ-inclusive school policies in our regression models, we helped rule out the possibility that the effects we observed were actually due to other key LGBTQ-supportive school interventions, suggesting that Out in Schools events may have had a small contributing relationship to better outcomes among students in schools where the events were held; we were also not able to identify whether the Out in Schools events occurred before or after the establishment of any GSAs or policies. In future research it would be valuable to incorporate information on whether or not Out in Schools presentations were hosted before or after the implementation of a GSA or anti-bullying policy, to better control for the timing of multiple interventions.

The present analyses only assessed the relationship between Out in Schools and the experiences of LGB and completely heterosexual students. We could not include gender minority (transgender, gender non-binary, or Two Spirit) students in this analysis, despite Out in Schools’ mandate of addressing transphobia and gender minority experiences as well. Fewer than 1% of the 2013 BCAHS sample self-identified as transgender, and a much larger proportion of students indicated they did not know what transgender meant, raising concerns about the reliability of the specific measure [40]. Research has shown that gender minority students experience similar health and social disparities as sexual minority students [41,42] but there is still limited research on the potential beneficial effects of these school-based interventions for gender minority students [43]. An important direction for future research would include exploring the effects of these school-based interventions for transgender and non-binary adolescents.

In addition to the limitations to be considered, this research also has some key strengths that should be noted. First, it draws on rigorously conducted, independently gathered, population-based data from across a large Canadian province, from nearly one thousand sexual minority adolescents and 20,000+ heterosexual students, to help evaluate the program. Rather than examining effects solely among youth who participated in the Out in Schools events within schools who had invited the program to present to the students, this quasi-experimental study design included a comparison group of students in schools that had never hosted the events, and we also were able to control for some key potential confounding variables. This study evaluates an intervention for promoting safer, more inclusive schools that has not been previously evaluated, as far as we could determine, nor has it been described in studies that have monitored multiple types of school-based interventions to support LGBTQ youth [44]. As such, this offers an additional new intervention for schools to consider, and preliminary evidence that it may have merit.

## 5. Conclusions

In sum, we found Out in Schools media-based events appeared to contribute to reduced odds of bullying, sexual orientation-based discrimination, and serious suicidal thoughts in the past year, as well as improving school connectedness among LGB students, regardless of gender, and reduced odds of discrimination, bullying, and suicidal thoughts for heterosexual students (primarily girls), with a possible additive dose-response relationship. These results were observed even in schools that also had a GSA or LGBTQ-inclusive policy, after controlling for the presence of these other interventions. Film-based interventions like Out in Schools’ program may serve as supplementary interventions to address bullying, further reduce homophobia, and foster inclusion in schools. Schools should be encouraged that these brief programs may offer an additional benefit as part of their overall strategies, ultimately improving their school climate, and the health and wellbeing of both LGB and heterosexual students.

## Figures and Tables

**Table 1:** Films shown by Out in Schools in their events during 2007 through 2013 *.

Titles of Films Shown	Years Shown	Top 10 Films with 30+ Showings
A Girl Named Kai	2008, 2009, 2010, 2011, 2012	9
Banana Boy	2007, 2008	
Benny’s Gym	2009	
Boys and Girls	2012, 2013	
Hello, My Name is Herman	2007, 2008, 2009, 2010, 2011, 2012, 2013	3
Hip Hop Homos	2008	
How Do I Say This I’m Gay	2010, 2011, 2012, 2013	7
I Don’t Want to Go Back Alone	2012, 2013	
Invisible Son	2007	
I Want to Know What It’s Like	2011, 2012, 2013	6
Laugh at Me	2008, 2009	
Leftovers	2007	
Love Exiled	2010, 2011	
My First Time Driving	2009	
No Bikini	2008, 2009, 2011, 2012	
Obama It Gets Better Video	2013	
Only Fags Listen to Pop Music	2011, 2012, 2013	5
Peking Turkey	2007, 2008, 2009, 2010, 2011, 2012, 2013	2
Prom Night	2007, 2008, 2009, 2010	
PSA Films Created by Students	2009, 2010, 2011, 2012, 2013	1
Rock Pockets	2009, 2010, 2011, 2012, 2013	5
Sissy Frenchfry	2007, 2008, 2009, 2010, 2011, 2012	8
Small Town Boy	2008, 2009	
The Queen	2011, 2012, 2013	10
Thicker than Water	2007	
To This Day	2012, 2013	
We Belong	2007, 2008, 2009, 2010, 2011, 2012	4
You are the Light of My Life	2009	
100% Woman	2008, 2009, 2011	
2 Spirits	2011, 2012, 2013	
50 Shades of Gay	2013	

* Films from years 2004 through 2006 unavailable.

**Table 2:** Lesbian, gay, bisexual and completely heterosexual student samples in the 2013 BC Adolescent Health Survey (BCAHS), unweighted *N* = 21,075, weighted and scaled * *N* = 184,821.

BCAHS Variable	Sample %	
	LGB	HET
Gender		
Boys	33.6%	51.5%
Girls	66.4%	48.5%
School Location		
Urban	89.6%	90.1%
Rural	10.4%	9.9%
Grade Level		
8	10.8%	18.0%
9	16.7%	19.0%
10	23.6%	20.1%
11	21.1%	21.2%
12	27.7%	21.7%
Attended Out in Schools Host School		
Yes	32.6%	69.1%
No	67.4%	30.9%

* Data weighted to adjust for differential probability of selection and scaled to provincial enrolment.

**Table 3:** Relationship between health outcomes among lesbian, gay, and bisexual and heterosexual students and whether a school has ever hosted an Out in Schools event.

**BCAHS Outcome**	**Lesbian, Gay, and Bisexual Students**			
	**Odds Ratios (95% CI)**			
	Unadjusted		Adjusted ^1^	
Discriminated Against Based on Sexual Orientation				
Boys	0.61 (0.37, 1.00)		0.64 (0.38, 1.08)	
Girls	**0.58 (0.40, 0.84)** *		**0.64 (0.44, 0.95)** *	
Seriously Considered Suicide				
Boys	0.69 (0.40, 1.18)		0.62 (0.36, 1.08)	
Girls	**0.57 (0.40, 0.82)** *		**0.62 (0.43, 0.90)** *	
Teased/Harassed in the Past Year				
Boys	**0.51 (0.32, 0.83)** *		**0.54 (0.33, 0.89)** *	
Girls	**0.67 (0.46, 0.96)** *		0.71 (0.49, 1.03)	
Excluded in the Past Year				
Boys	0.76 (0.47, 1.21)		0.76 (0.46, 1.26)	
Girls	0.76 (0.52, 1.10)		0.82 (0.55, 1.21)	
	**Regression Coefficients (95% CI)**			
		**R^2^ Value**		**R^2^ Value**
School Belonging Score				
Boys	−0.15 (−0.37, 0.07)	0.006	−0.16 (−0.39, 0.07)	0.027
Girls	**−0.15 (−0.29, −0.01)** *	0.006	−0.11 (−0.25, 0.03)	0.030
Teacher Connectedness Score				
Boys	−0.12 (−0.31, 0.08)	0.004	−0.11 (−0.31, 0.08)	0.031
Girls	−0.10 (−0.22, −0.02)	0.003	−0.10 (−0.22, 0.02)	0.017
**BCAHS Outcome**	**Completely Heterosexual Students**			
	**Odds Ratios (95% CI)**			
	Unadjusted		Adjusted ^1^	
Discriminated Against Based on Sexual Orientation				
Boys	0.98 (0.75, 1.29)		1.07 (0.59, 1.95)	
Girls	0.71 (0.48, 1.06)		0.75 (0.50, 1.15)	
Seriously Considered Suicide				
Boys	0.96 (0.81, 1.15)		0.95 (0.79, 1.14)	
Girls	**0.82 (0.71, 0.95)** *		**0.83 (0.72, 0.97)** *	
Teased/Harassed in the Past Year				
Boys	0.93 (0.84, 1.03)		0.95 (0.86, 1.06)	
Girls	**0.89 (0.81, 0.97)** *		0.92 (0.84, 1.01)	
Excluded in the Past Year				
Boys	1.05 (0.94, 1.17)		1.05 (0.94, 1.17)	
Girls	**0.85 (0.77, 0.94)** *		**0.88 (0.79, 0.97)** *	
	**Regression Coefficients (95% CI)**			
		**R^2^ Value**		**R^2^ Value**
School Belonging Score				
Boys	0.02 (−0.02, 0.06)	<0.001	0.01 (−0.03, 0.05)	0.010
Girls	0.01 (−0.04, 0.05)	<0.001	−0.002 (−0.04, 0.04)	0.007
Teacher Connectedness Score				
Boys	**0.04 (0.001, 0.07)** *	0.001	0.03 (−0.01, 0.06)	0.007
Girls	**0.06 (0.03, 0.10)** *	0.002	**0.05 (0.01, 0.09)** *	0.009

^1^ Adjusted for presence of GSA/anti-homophobia school policy, grade level and urban/rural school location. * Statistically significant (*p* < 0.05).

**Table 4:** Relationship between health outcome measures among lesbian, gay, bisexual and heterosexual students and number of Out in Schools events hosted per school since 2004.

**BCAHS Outcome**	**Lesbian, Gay, and Bisexual Students**			
	**Odds Ratios (95% CI)**			
	Unadjusted		Adjusted ^1^	
Discriminated Against Based on Sexual Orientation				
Boys	**0.83 (0.71, 0.96)** *		**0.84 (0.72, 0.98)** *	
Girls	**0.90 (0.82, 1.00)** *		0.93 (0.83, 1.04)	
Seriously Considered Suicide				
Boys	0.90 (0.76, 1.08)		0.89 (0.74, 1.07)	
Girls	**0.85 (0.76, 0.94)** *		**0.86 (0.78, 0.96)** *	
Teased/Harassed in the Past Year				
Boys	**0.83 (0.72, 0.95)** *		**0.84 (0.74, 0.96)** *	
Girls	**0.90 (0.83, 0.99)** *		0.92 (0.84, 1.01)	
Excluded in the Past Year				
Boys	**0.84 (0.73, 0.96)** *		**0.84 (0.73, 0.97)** *	
Girls	0.92 (0.84, 1.02)		0.95 (0.86, 1.05)	
	**Regression Coefficients (95% CI)**			
		**R^2^ Value**		**R^2^ Value**
School Belonging Score				
Boys	0.04 (−0.02, 0.11)	0.007	0.04 (−0.02, 0.11)	0.027
Girls	**0.04 (0.01, 0.08)** *	0.007	0.03 (−0.003, 0.07)	0.031
Teacher Connectedness Score				
Boys	**0.05 (0.00, 0.09)** *	0.009	**0.05 (0.001, 0.09)** *	0.037
Girls	0.02 (−0.002, 0.05)	0.003	0.02 (−0.004, 0.05)	0.016
**BCAHS Outcome**	**Completely Heterosexual Students**			
	**Odds Ratios (95% CI)**			
	Unadjusted		Adjusted ^1^	
Discriminated Against Based on Sexual Orientation				
Boys	1.03 (0.96, 1.10)		1.03 (0.97, 1.10)	
Girls	**0.84 (0.74, 0.95)** *		**0.85 (0.74, 0.97)** *	
Seriously Considered Suicide				
Boys	0.99 (0.94, 1.04)		0.98 (0.93, 1.03)	
Girls	**0.95 (0.91, 0.98)** *		**0.95 (0.92, 0.98)** *	
Teased/Harassed in the Past Year				
Boys	0.99 (0.97, 1.01)		1.00 (0.98, 1.02)	
Girls	**0.97 (0.95, 0.99)** *		0.98 (0.96, 1.00)	
Excluded in the Past Year				
Boys	1.01 (0.98, 1.03)		1.01 (0.98, 1.03)	
Girls	**0.97 (0.95, 0.99)** *		**0.98 (0.96, 0.997)** *	
	**Regression Coefficients (95% CI)**			
		**R^2^ Value**		**R^2^ Value**
School Belonging Score				
Boys	−0.01 (−0.01, 0.002)	0.001	−0.01 (−0.01, 0.002)	0.010
Girls	−0.01 (−0.02, 0.01)	<0.001	−0.004 (−0.01, 0.01)	0.007
Teacher Connectedness Score				
Boys	**−0.01 (−0.02, −0.01)** *	0.001	**−0.01 (−0.02, −0.003)** *	0.008
Girls	**−0.02 (−0.03, −0.01)** *	0.003	**−0.02 (−0.02, 0.01)** *	0.011

^1^ Adjusted for presence of GSA/anti-homophobia school policy, grade level and urban/rural school location. * Statistically significant (*p* < 0.05).

**Table 5:** Relationship between BC Adolescent Health Survey health outcome measures and the number of Out in Schools events hosted per school during the 2013 BCAHS cycle (2009–2013), for lesbian, gay, bisexual and completely heterosexual students.

**BCAHS Outcome**	**Lesbian, Gay, and Bisexual Students**			
	**Odds Ratios (95% CI)**			
	Unadjusted		Adjusted ^1^	
Discriminated Against Based on Sexual Orientation				
Boys	0.87 (0.72, 1.06)		0.89 (0.73, 1.08)	
Girls	0.88 (0.77, 1.01)		0.92 (0.80, 1.06)	
Seriously Considered Suicide				
Boys	0.95 (0.76, 1.19)		0.95 (0.75, 1.20)	
Girls	**0.82 (0.72, 0.93)** *		**0.84 (0.74, 0.95)** *	
Teased/Harassed in the Past Year				
Boys	**0.82 (0.67, 1.00)** *		0.84 (0.68, 1.02)	
Girls	**0.89 (0.80, 0.99)** *		0.92 (0.82, 1.03)	
Excluded in the Past Year				
Boys	0.83 (0.69, 1.00)		0.83 (0.67, 1.01)	
Girls	0.90 (0.80, 1.01)		0.92 (0.82, 1.04)	
	**Regression Coefficients (95% CI)**			
		**R^2^ Value**		**R^2^ Value**
School Belonging Score				
Boys	0.06 (−0.04, 0.15)	0.005	0.05 (−0.04, 0.15)	0.025
Girls	**0.04 (0.002, 0.08)** *	0.005	0.03 (−0.01, 0.07)	0.029
Teacher Connectedness Score				
Boys	**0.08 (0.02, 0.14)** *	0.014	**0.08 (0.02, 0.14)** *	0.042
Girls	0.03 (−0.002, 0.06)	0.003	0.03 (−0.01, 0.06)	0.015
**BCAHS Outcome**	**Completely Heterosexual Students**			
	**Odds Ratios (95% CI)**			
	Unadjusted		Adjusted ^1^	
Discriminated Against based on Sexual Orientation				
Boys	1.01 (0.92, 1.11)		1.02 (0.93, 1.12)	
Girls	0.87 (0.74, 1.02)		0.88 (0.75, 1.04)	
Seriously Considered Suicide				
Boys	0.97 (0.91, 1.04)		0.97 (0.91, 1.03)	
Girls	**0.93 (0.89, 0.98)** *		**0.94 (0.89, 0.99)** *	
Teased/Harassed in the Past Year				
Boys	0.99 (0.96, 1.02)		0.99 (0.96, 1.03)	
Girls	**0.97 (0.94, 0.998)** *		0.98 (0.95, 1.01)	
Excluded in the Past Year				
Boys	1.00 (0.96, 1.04)		1.00 (0.96, 1.03)	
Girls	**0.96 (0.94, 0.99)** *		0.97 (0.94, 1.00)	
	**Regression Coefficients (95% CI)**			
		**R^2^ Value**		**R^2^ Value**
School Belonging Score				
Boys	−0.01 (−0.02, 0.001)	<0.001	−0.01 (−0.02, 0.003)	0.010
Girls	−0.004 (−0.02, 0.01)	<0.001	−0.003 (−0.02, 0.01)	0.007
Teacher Connectedness Score				
Boys	**−0.02 (−0.03, −0.01) ^2,^** *	0.001	**−0.02 (−0.03, −0.01) ^2,^** *	0.008
Girls	**−0.02 (−0.03, −0.01) ^2,^** *	0.002	**−0.02 (−0.03, −0.01) ^2,^** *	0.010

^1^ Adjusted for presence of GSA/anti-homophobia school policy, grade level and urban/rural school location. ^2^ The estimates and CI were different in the third decimal place. * Statistically significant (*p* < 0.05).
